# Retro-orbital injection of FITC-dextran combined with isolectin B4 in assessing the retinal neovascularization defect

**DOI:** 10.1186/s12886-021-01969-5

**Published:** 2021-05-11

**Authors:** Jizhu Li, Yuqing Wu, Bingqian Liu, Ying Huang, Qingxiu Wu, Haichun Li, Sainan Xiao, Ying Lin, Tao Li

**Affiliations:** State Key Laboratory of Ophthalmology, Zhongshan Ophthalmic Center, Sun Yat-sen University, Jinsui Road 7, Tianhe District, 510000 Guangzhou, China

**Keywords:** Retro‐orbital injection, Isolectin B4, FITC-dextran, Neovascularization, Retina, Vitreous blood vessel

## Abstract

**Background:**

A reliable and effective method is required to deliver agent that can aid the in vivo imaging of retinal vessels. The aim of the present study was to evaluate retro-orbital (RO) injection of fluorescein-labeled isothiocyanate dextran (FITC-dextran) as a method of demonstrating retinal neovascularization (NV) and avascular areas in oxygen-induced retinopathy (OIR) mice.

**Methods:**

Different concentrations of FITC-dextran were used to compare the efficacy of this agent in perfusing the retinal vessels. Hematoxylin–eosin (HE) staining was used to evaluate the safety of RO injection. The vitreous blood vessels and extent of NV were assessed in P17 OIR mice using FITC-dextran and compared with the corresponding measurements obtained following isolectin B4 staining or the combination of both methods.

**Results:**

The fluorescence of small vessels and neovascular tufts could be observed clearly following RO injection of 0.05 ml of 25 mg/ml or 50 mg/ml FITC-dextran. No visible damage to tissues adjacent to the injection site was discovered. Vitreous blood flow was gradually reduced from P0 to P5 and eventually disappeared in P17 OIR mice, as demonstrated by FITC-dextran perfusion. The retinal NV areas assessed by isolectin B4 were larger than those assessed by FITC-dextran, but the retinal avascular areas were smaller. The combination of both methods could conduce to distinguish non-functional blood vessels.

**Conclusions:**

RO injection of FITC-dextran combined with isolectin B4 is an effective, optimal method for assessing the NV area and avascular area.

## Background

Retinal neovascularization (NV) is a severe complication of various diseases, such as diabetic retinopathy, central retinal vein occlusion and retinopathy of prematurity (ROP) [1–3]. Animal models are usually used to study the pathogenesis of disease and develop effective treatments of retinal NV. One of the well-established animal models is the oxygen-induced retinopathy (OIR) mouse model, which offers a repetitive platform for studying retinal NV [4–6]. The extent of retinal NV is usually evaluated in vivo to facilitate the identification of a reliable and effective method to deliver agents used for the visualization of retinal vessels. The tail vein injection is one of the most commonly used administration routes of agents, but it is technically challenging and frequently exhibits a high rate of failure [7–10]. An alternative method is intraperitoneal injection, which is also not optimal since it cannot prevent the first-pass metabolism noted in the liver [11, 12]. Therefore, additional time is required for successful retinal perfusion. Furthermore, intraperitoneal injection requires a higher dose of fluorescein-labeled isothiocyanate dextran (FITC-dextran), which can cause additional damage.

Retro-orbital (RO) injection in mice has been proven to be a simple and optimal method with regard to delivery [13, 14]. We have previously shown that RO injection of FITC-dextran is an effective and reliable method for observing mouse retinal vessels [15]. The agents used for RO injection can prevent the first-pass metabolism occurring in the liver [12]. Although previous studies demonstrated that the agents could be absorbed by the blood circulatory system [16–18], the principle and mechanism of RO injection in mice remain elusive.

Various agents, such as FITC-dextran, CD31 and isolectin B4 [19–21], can be used to visualize retinal blood vessels. However, only a limited number of reports have compared the efficacy of different agents in visualizing the retinal vessels.

The present study aimed to determine the principle of RO injection in mice and its potential in distinguishing the vitreous body or retinal blood vessels of mice and/or evaluating the extent of the NV and the avascular areas.

## Methods

### Animals

C57BL/6J mouse were purchased from the breeding facility at GemPharmatech. All experiments adhered to the ARVO statement for the use of animals in ophthalmic and vision research and were approved by the Animal Care and Use Committee of the Zhongshan Ophthalmic Centre at the Sun Yat-sen University of Guangzhou in China. The OIR C57BL/6J mouse model was established according to a previously described method [22]. On postnatal day 7 (P7), mouse pups and their nursing dams were exposed to 75 ± 2 % oxygen in a hyperoxia chamber until P12. The mice were then exposed to room air. The maximum retinal NV of the OIR mice occurred at P17. The nursing mothers were rotated with the surrogate mother from high oxygen to room air every 24 h to prevent oxygen toxicity.

### Different levels of FITC-Dextran

The mice were anesthetized with an intraperitoneal injection of 50 mg/kg pentobarbital and placed in right lateral recumbency with their heads facing to the left. A gentle pressure of two fingers was applied on the peri-orbital area, allowing the easiest administration of the injection into the left RO sinus of the animal. A 27-gauge needle was used at a 45° angle to produce a puncture of 2–3 mm in length into the mouse’s orbital venous sinus. To assess the efficacy of the FITC-dextran injection, P17 OIR mice were injected with different concentrations (50, 25 and 12.5 mg/ml) of 0.05 ml FITC-dextran.

### Orbital tissue perfusion

To investigate whether FITC-dextran was absorbed into the mouse blood circulation system, we performed RO injection of 0.05 ml FITC-dextran (50 mg/ml) in anesthetized P17 OIR mice (50 mg/kg pentobarbital). After mouse euthanization by CO2 inhalation, the eyes and orbital tissues were removed, placed on a glass slide and imaged by fluorescence microscopy separately (Zeiss Axioplan 2 Imaging; Zeiss, Oberkochen, Germany).

### Histological evaluation staining

To examine whether the morphology of the orbital tissues was damaged by RO injection, we performed RO injection with 0.10 ml of phosphate-buffered saline (PBS) in 5 anesthetized P0 mouse (50 mg/kg pentobarbital), and then eyes were enucleated after euthanization (CO2 inhalation). The samples were placed in a liquid mixture containing 1 % formaldehyde (Sigma-Aldrich, St. Louis, MO, USA) and 1.25 % glutaraldehyde (Sigma-Aldrich) for at least 48 h and embedded in paraffin. Serial Sec. (4 μm thick) were cut in a sagittal plane through the cornea, retina, orbital venous sinus and surrounding tissues. The tissue sections were stained with hematoxylin and eosin (HE). Only the sagittal sections, which were cut through the orbital venous sinus and the surrounding tissue, were selected. The images were visualized using a microscope (Zeiss Axioplan 2 Imaging; Zeiss, Oberkochen, Germany).

### Isolectin B4 staining combined with FITC-Dextran perfusion

To evaluate the vitreous body vasculature of the mice, we perfomed RO injection of FITC-dextran in 15 normal postnatal mice (P1, P3 and P5) and 5 OIR mice (P17) after anesthetization (50 mg/kg pentobarbital), then eyes were enucleated after euthanization (CO2 inhalation). The samples were placed in 4 % paraformaldehyde for 40 min at room temperature. The cornea, iris and lens were gently removed using a stereomicroscope (Mz6; Leica, Mannheim, Germany). The retinas were incubated in PBS containing 2 % isolectin B4 (Alexa Fluor 568; Invitrogen, Carlsbad, CA, USA) overnight at 4 °C. The retinas stained with isolectin B4 were washed with PBS, and subsequently, retinal flat mounts were made.

### Retinal flat mounts

Four radial incisions were made in the dissected retina, which was flattened by a coverslip. Retinal flat mounts were kept in the dark and imaged by fluorescence microscopy. Retinal segments were merged into a total retinal image (Photoshop CS4; Adobe Systems, Inc., San Jose, CA, USA).

### Quantification of the NV or avascular areas

To study the retinal NV or avascular rear areas, we stained 15 P17 OIR retinas with isolectin B4, FITC-dextran or a combination of isolectin B4 with FITC-dextran. The samples were subsequently imaged separately by fluorescence microscopy using red, blue or hybrid exciting light. Retinal NV and avascular areas as well as the total retinal area were measured and analyzed using Image Pro-Plus 5.1 software (Media Cybernetics, Inc., Rockville, MD, USA).

### Statistical analysis

Statistical analysis was performed using the SPSS software version 16.0 (SPSS, Inc., Chicago, IL, USA). Data are reported as the mean ± SD. ANOVA was used for multiple-group comparisons. A *P* value less than 0.05 (*P* < 0.05) was considered significant.

## Results

### Principle of RO injection

**Fig. 1:**
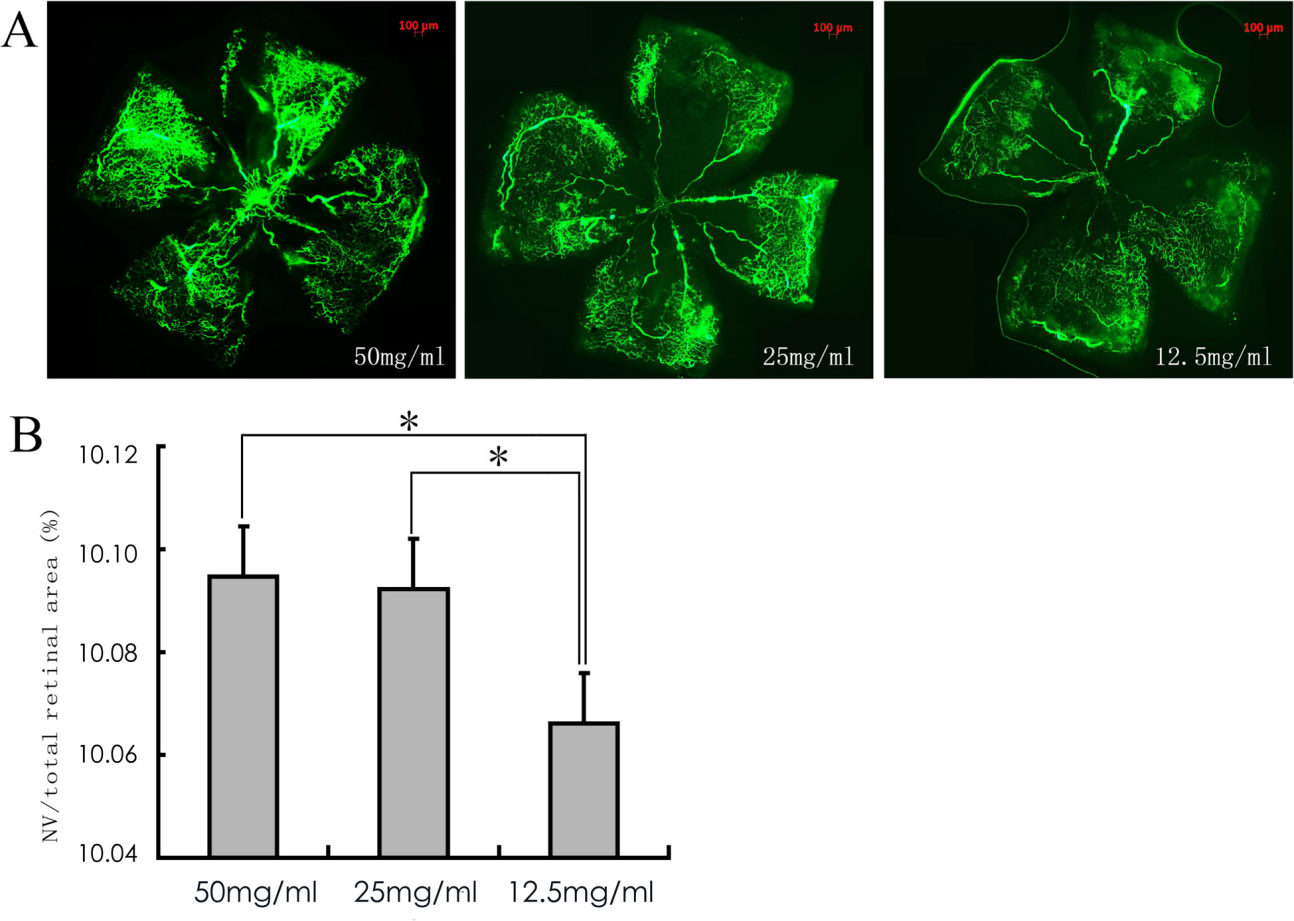
The effects of different concentrations of FITC-dextran in P17 OIR mice following retro-orbital (RO) injection. **a**: The fluorescence of small vessels and neovascular tufts following administration of 0.05 ml of 12.5 mg/ml FITC-dextran was too weak, whereas these structures were fully observed in the presence of 0.05 ml of 25 mg/ml and/or 50 mg/ml FITC-dextran. Original magnification, 50×; **b**: Quantification of retinal neovascularization (NV) in P17 OIR mice among the three groups. Compared to 12.5 mg/ml FITC-dextran, 25 mg/ml and 50 mg/ml FITC-dextran showed a significant increase in the NV/total retinal area% (*P* < 0.05). (*P < 0.05, **b**) *n* = 9 from 9 mice

The fluorescence imaging data of small vessels and neovascular tufts were fully observed as determined by RO injection of 0.05 ml (25 mg/ml or 50 mg/ml) FITC-dextran in the P17 OIR mice, while the fluorescence was too weak at the concentration of 12.5 mg/ml (Fig. 1a).

**Fig. 2:**
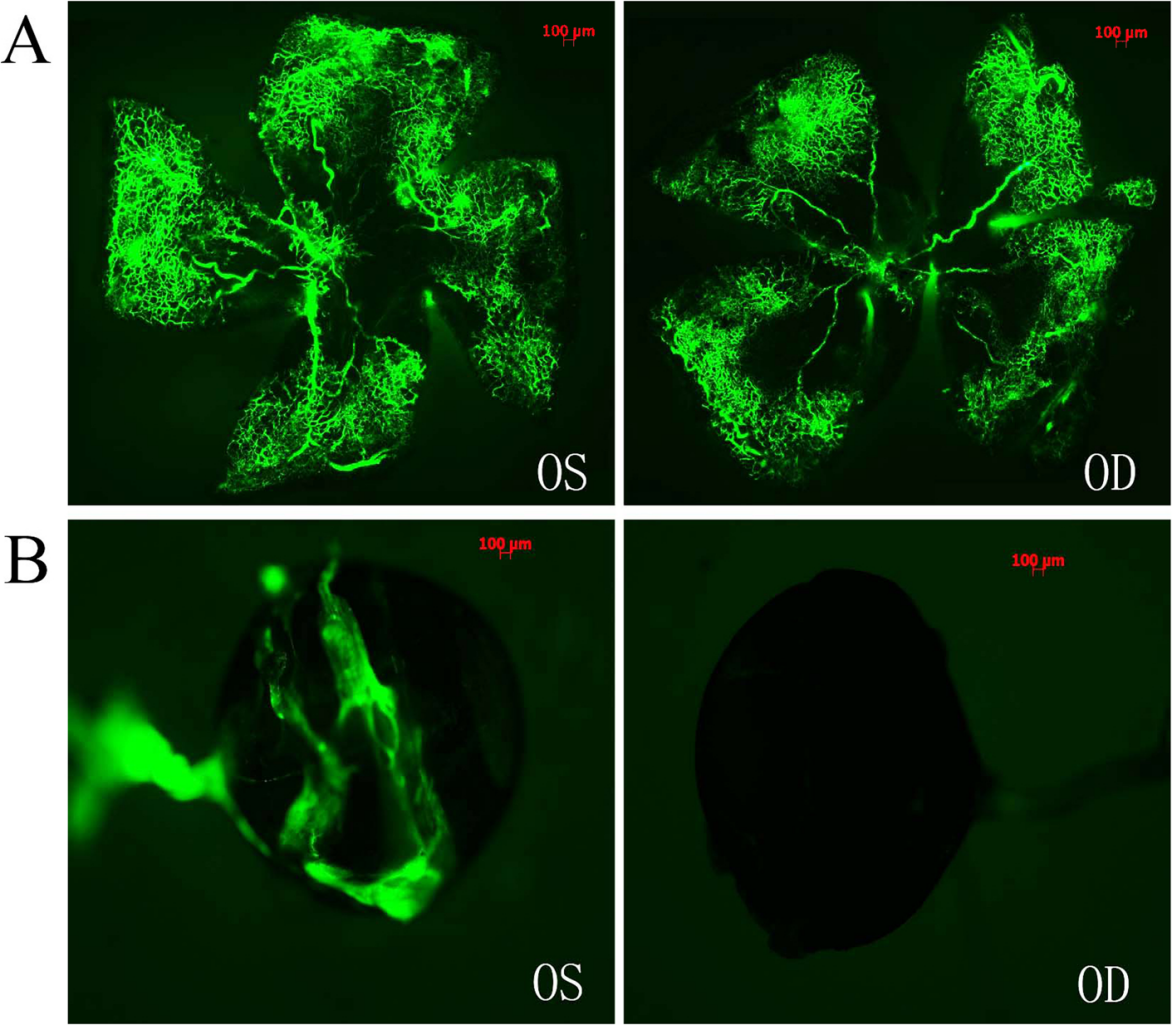
The fluorescence intensity of retina and orbital tissues in P17 OIR mice following retro-orbital (RO) injection. **a**: The fluorescence intensity of the retinal images of the uninjected eye (OD) was the same as that of the injected eye (OS); **b**: The orbital tissues of the injected eyes were perfused with FITC-dextran using the retro-orbital (RO) injection in P17 OIR mice. The surrounding orbital tissue of the injected eye (OS) represented high fluorescence intensity, and no fluorescence was detected in the uninjected eye (OD). *n* = 5 from 5 mice. Original magnification, 50×

**Fig. 3:**
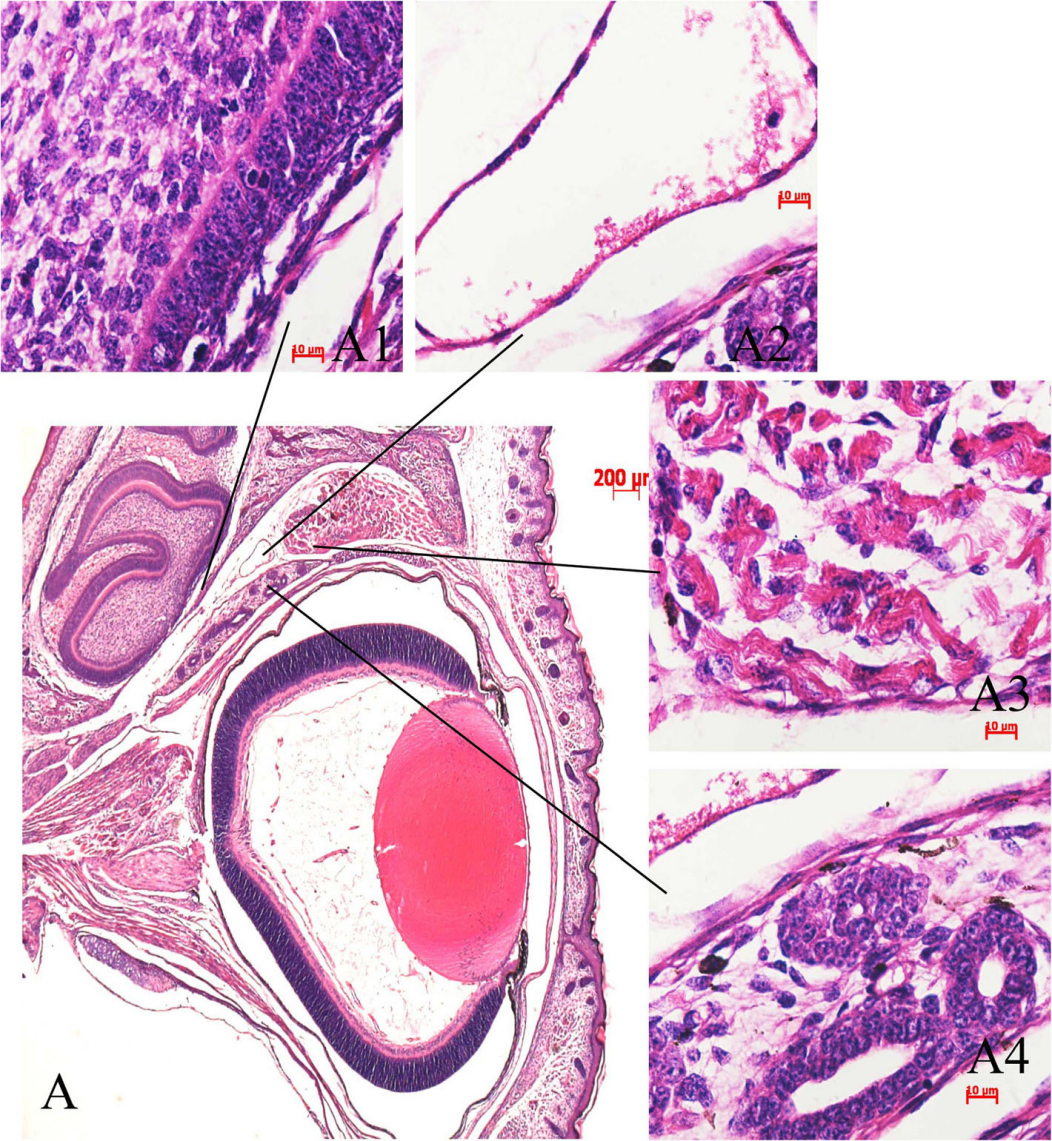
Histologic section of the injected eyes of the P0 mice following retro-orbital (RO) injection of 0.10 ml of PBS. The insets indicate that the eye and the surrounding tissue (**a**), including the forebrain (**a1**), orbital vessel sinus (**a2**), eye muscle (**a3**) and Harderian gland (**a4**), appear normal. *n* = 5 from 5 mice. A, original magnification 25×. A1–A4, original magnification, 1,000×

A portion of FITC-dextran leaked from the orbital tissues of the injected eyes. However, this leakage was not noted in the orbital tissues of the uninjected eyes (Fig. 2b). The surrounding orbital tissues include the optic nerve, extraocular muscles, fascias, conjunctiva and sclera. After retro-orbital (RO) injection of FITC-dextran, the surrounding orbital tissues of the injected eye (OS) were perfused because of diffusion, which represented high fluorescence intensity, while no fluorescence was detected in the surrounding orbital tissues of the uninjected eye (OD). We further observed that the fluorescence intensity of the retinal images of the injected eyes was the same as that of the uninjected eyes (Fig. 2a), probably because after retro-orbital (RO) injection of FITC-dextran, the concentration of FITC-dextran diffused into blood circulation through the orbital venous sinus, and fluorescence passed through the blood circulation system into both eyes. The postmortem histological samples derived from the P0 mice that received RO injection of 0.10 ml PBS indicated that the tissues around the orbital venous sinus, such as the Harderian gland, the muscle and forebrain, were not damaged by the injection (Fig. 3).

### Vitreous body blood vessel vegression

**Fig. 4:**
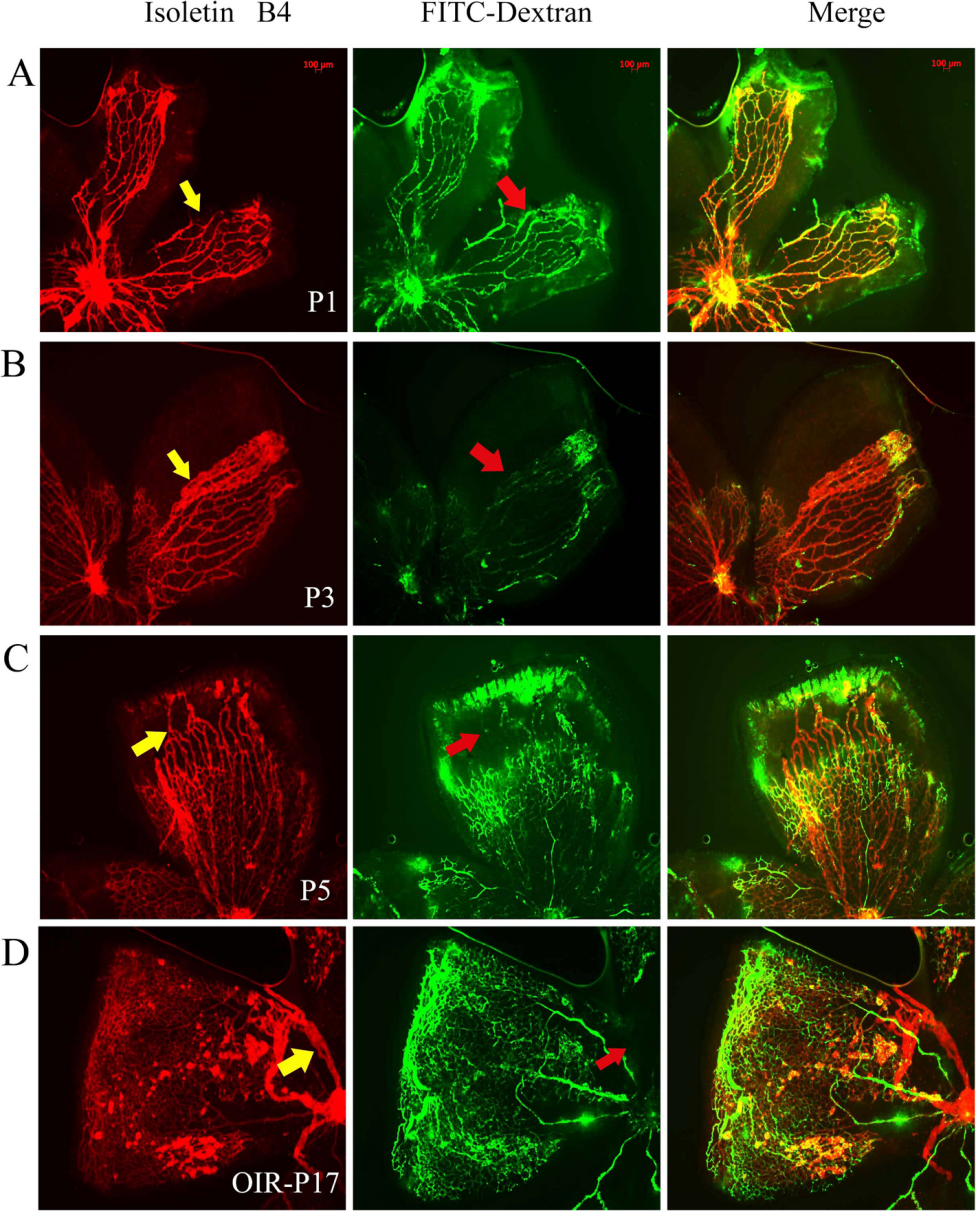
Vitreous body vessels regressed in mice. The vessels were stained with isolectin B4 alone, FITC alone, and isolectin B4 combined with FITC-dextran by retro-orbital (RO) injection. **a**: Fluorescence of the vitreous body vessels filled with FITC-dextran (red arrow) or stained with isolectin B4 (yellow arrow) from the P1 samples; **b**: On P3, the FITC-dextran fluorescence of the vitreous body vessels was slightly reduced (red arrow); **c**: On P5, the FITC-dextran fluorescence of specific vitreous body vessels was considerably reduced (red arrow); **d**: The vitreous body vessels (yellow arrow) of OIR-P17 mice could not be fully injected with FITC-dextran. *n *= 20 from 20 mice. Original magnification, 50×

The fluorescence images indicated that the vitreous body blood vessels of the neonatal mice could be visualized by RO injection of FITC-dextran. The vitreous body blood vessels stained with isolectin B4 revealed uniform red images, and the vitreous body blood vessels perfused with FITC-dextran presented nonuniform green images. In P1 mice, the peripheral small vitreous body vessels that were stained with isolectin B4 could also be perfused with FITC-dextran. In P3 mice, the peripheral small vitreous vessels stained with isolectin B4 could only be partially perfused using FITC-dextran. In P5 mice, the peripheral small vitreous body vessels stained with isolectin B4 could not be perfused with FITC-dextran (Fig. 4a–c). In P17 OIR mice, a part of the central large vitreous body vessel area stained with isolectin B4 could not be perfused further with FITC-dextran (Fig. 4d). Altogether, the results suggested that the blood flow in the vitreous body vessels was restricted prior to the regression of the vessels.

### Quantification of the retinal NV or the avascular areas

The retinal NV areas (presented as a percentage of the retinal NV area in the total retinal area) visualized by different concentrations of FITC-dextran (50, 25 and 12.5 mg/ml) were compared. A significant decrease was found in the 12.5 mg/ml FITC-dextran group (0.10066 ± 0.00008) (*P* < 0.05). No significant difference in the retinal NV area was noted between the 50 mg/ml FITC-dextran (0.10095 ± 0.00012) and 25 mg/ml FITC-dextran (0.10092 ± 0.00009) (*P* > 0.05) groups (Fig. 1b).

**Fig. 5:**
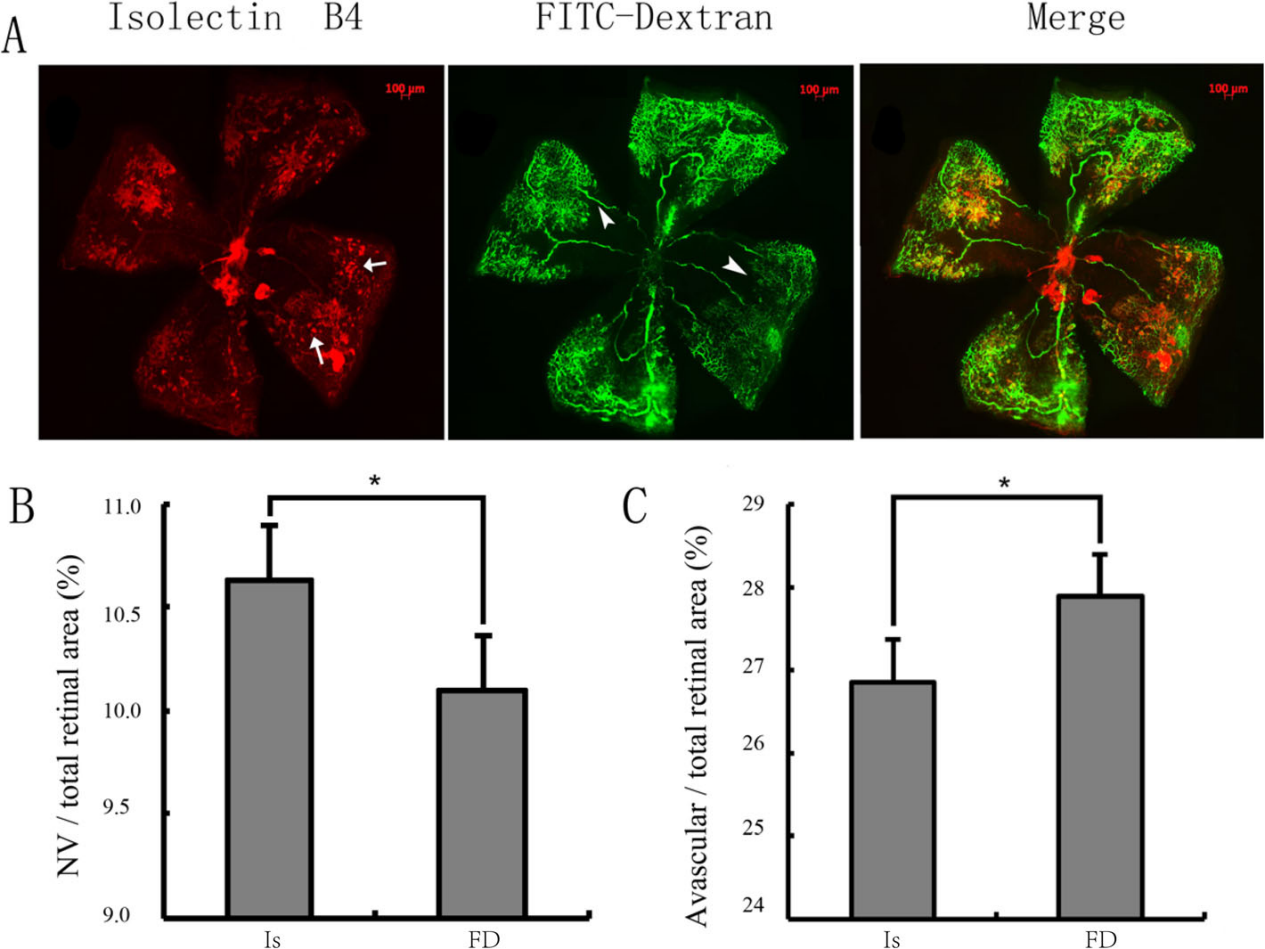
Quantification of the retinal neovascularization (NV) and avascular areas in P17 OIR mice. **a**: The retinal blood vessels were stained with isolectin B4 alone, FITC alone, and a combination of isolectin B4 and FITC-dextran. The remaining vitreous body vessels (arrow) and the unfilled small retinal vessels (arrow bead) were taken into consideration with regard to the estimation of the retinal NV or avascular areas. Original magnification, 50×; **b** and **c**: The retinal NV and avascular areas were evaluated by administration of isolectin B4 alone and FITC-dextran alone. *n* = 5 from 5 mice. *P < 0.05. The two groups were compared

The retinal NV and avascular areas visualized by FITC-dextran perfusion and IB4 staining were also compared. Most of the NV areas showed positive FITC-dextran perfusion, which suggested that those NV support circulatory function. While adjacent to avascular areas, some blood vessels were only stained by IB4 without clearly FITC-dextran perfusion, suggesting that these vessels may be non-functional (5 A). The retinal NV areas evaluated by FITC-dextran only (0.1009 ± 0.001) exhibited weaker staining than those evaluated by isolectin B4 only (0.1049 ± 0.002) (*P* < 0.01) (Fig. 5b). The retinal avascular areas evaluated by FITC-dextran only (0.2850 ± 0.001) were larger than those evaluated by isolectin B4 only (0.2676 ± 0.002) (*P* < 0.01) (Fig. 5c).

## Discussion

Previous studies have shown that RO injection is a simple method applied in mice that could be used to deliver agents for the imaging of retinal vessels [23]. Compared with intraperitoneal injection, RO injection exhibits the following advantages: first, the agents of retro-orbital injection can avoid the first-pass metabolism occurring in the liver [12]. Second, this process requires less time to achieve retinal perfusion. Following retro-orbital injection, FITC-dextran diffuses into the orbital venous sinus and blood circulation very rapidly. Our previous studies have shown that only 3 s are required to accomplish retinal perfusion[15]. Third, a smaller dose (0.05 ml) of FITC-dextran was required for retro-orbital injection, which could effectively reduce the incidence of allergic reactions and mortality, as well as the experimental costs. The use of retro-orbital injection caused less systemic reactions due to a smaller injection dose[23], whereas no severe allergic reaction or mortality was reported in our experiment. Fourth, all the technicians who participated in the present study were ophthalmologists who were familiar with the orbital anatomical structure. The performance of retro-orbital and intraperitoneal injections is relatively easy. The procedure of retro-orbital injection is described in the manuscript.

However, the mechanism by which injected FITC-dextran enters the retinal vasculature has not been fully clarified. In the present study, two main possible mechanisms were provided. Initially, the data showed that the FITC-dextran administered by the RO route could not be directly injected into the orbital venous sinus, which could be proven by the relevant histological findings indicating the absence of morphological changes in the orbital venous sinus of the injected eyes. In addition, safely elevated orbital pressure was required during RO injection. In the present study, the retinal fluorescence images of RO injection (0.05 ml of 25 mg/ml FITC-dextran) could be readily noted, whereas in our previous study, the retinal images of RO injection (0.04 ml of 50 mg/ml FITC-dextran) could not be readily distinguished [15]. These findings suggested that the injected volume of FITC-dextran may be more important than the concentration of the injected FITC-dextran. Furthermore, we speculated that orbital pressure may be temporarily increased by injection of FITC-dextran and that elevated orbital pressure could facilitate the diffusion of FITC-dextran into the orbital venous sinus and blood circulation.

In the present study, the morphology of the tissues around the injection site was not affected following RO injection of 0.10 ml PBS. We further demonstrated that RO injection of 0.05 ml FITC-dextran was sufficient to provide a clear visualization of the retinal vessels, and FITC-dextran could diffuse rapidly into the blood circulation, indicating that elevated orbital pressure noted following RO injection could be rapidly reduced. Therefore, we suggest that this type of retinal vessel imaging, which utilizes RO injection of FITC-dextran, is safe and effective.

Vitreous body blood vessels could be observed in the majority of the mouse pups, and they regressed with the development of the retinal vasculature [24]. However, previous studies that have investigated blood flow changes with regard to the regression of vitreous body blood vessels are limited. In the present study, we developed a method to study these changes using RO injection of FITC-dextran. We observed that the blood flow in the peripheral small vitreous vessels gradually decreased from P1 to P5 prior to vessel regression. A similar feature was observed in the central large vitreous vessels of the P17 OIR mice. These findings are consistent with a previous study indicating that the diameter of proximal vitreous arterial vasoconstriction preceded vessel regression [25].

Several studies have reported that isolectin B4 or CD31 combined with FITC-dextran can be used to evaluate the retinal NV and avascular area [26–28]. However, isolectin B4 can stain only retinal vessel endothelial cells and cannot be used to observe retinal blood flow. CD31 combined with FITC-dextran requires several complicated procedures. In the present study, we demonstrated that the retinal NV areas assessed by isolectin B4 were larger than those assessed by FITC-dextran, but the retinal avascular areas were smaller. A possible explanation for this finding could that some NV vessels or avascular area adjacent vessels were non-functional, which could be stained by isolectin B4 but could not be perfused by FITC-dextran. The decrease in blood flow in some of the retinal vessels could be similar to the blood flow changes noted in the regressed vitreous vessels. Furthermore, some small retinal blood vessels could be covered by the remaining vitreous body blood vessels or other substances [29]. Therefore, isolectin B4 staining can be used as a useful alternative method for FITC-dextran to provide a more accurate assessment of the retinal NV or the avascular areas. And RO injection of combined isolectin B4 with FITC-dextran provides an efficient and simple access to distinguish functional and non-functional blood vessels.

The stability of FITC-dextran in vitro and in vivo is optimal only at elevated pH (> 9), whereas at elevated pH levels, a considerable risk is present for hydrolysis of the fluorescein label [30]. FITC-dextran exhibits attenuation effects as noted for other compounds used for in vivo imaging. It is recommended to terminate in vivo imaging within 30 min following the injection. The following methods were used in the present study to minimize the attenuation of the fluorescent signal of FITC-dextran: prior to injection, all FITC-dextran and isolectin B4 injections were performed in the dark. The concentration of FITC-dextran decreased following sacrifice of the animals. Appropriate doses of FITC-dextran were administered by RO injection, and the eyes were subsequently enucleated to guarantee an adequate concentration of FITC-dextran in retinal blood vessels. The samples were incubated at 4 °C overnight. A temperature lower than 4 °C would delay the attenuation of FITC-dextran.

## Conclusions

In summary, the present study described the principle of RO injection by assessing the diffusion of FITC-dextran into the orbital venous sinus with temporarily elevated orbital pressure. Moreover, RO injection of FITC-dextran is a useful method for monitoring the blood flow of the vitreous body of mice. Isolectin B4 staining is an optimal alternative method to FITC-dextran in assessing retinal NV or avascular areas. The combination use of FITC-dextran and isolectin B4 by RO injection provoides an efficient access for distinguishing functional and non-functional blood vessels.

## Data Availability

The datasets used and analyzed during the current study are available from the corresponding author on reasonable request.

## Abbreviations

RO: Retro-orbital
FITC-dextran: Fluorescein-labeled isothiocyanate dextran
NV: Retinalneovascularization
OIR: Oxygen-induced retinopathy
HE: Hematoxylin–eosin
ROP: Retinopathy of prematurity
HE: Hematoxylin and eosin
